# The Use of *Sargassum* spp. Ashes Like a Raw Material for Mortar Production: Composite Performance and Environmental Outlook

**DOI:** 10.3390/ma17081785

**Published:** 2024-04-12

**Authors:** Gabriela Pitolli Lyra, Ana Letícia Colombo, Afonso José Felício Peres Duran, Igor Machado da Silva Parente, Cristiane Bueno, João Adriano Rossignolo

**Affiliations:** 1 Department of Biosystems Engineering, Faculty of Animal Science and Food Engineering, Universidade de São Paulo (USP), Pirassununga 13635-900, Brazil; gabriela.lyra@usp.br; 2 Post-Graduation Program in Material Science and Engineering, Faculty of Animal Science and Food Engineering, Universidade de São Paulo (USP), Pirassununga 13635-900, Brazil; analeticia.colombo@usp.br (A.L.C.); afonso.duran@usp.br (A.J.F.P.D.); igorparente@usp.br (I.M.d.S.P.); 3 Department of Civil Engineering, Universidade Federal de São Carlos (UFSCAR), São Carlos 13565-905, Brazil; cbueno@ufscar.br

**Keywords:** seaweed ash, cementitious composites, biomass, civil construction, LCA

## Abstract

The accumulation of brown algae from the genus *Sargassum* has been increasing over the years in coastal regions of the Caribbean, Africa, Brazil, and Mexico. This causes harmful effects to the ecosystem, human health, the economy, and the climate due to gas emissions from its decomposition process. There is the possibility of this biomass being reused in civil construction, and some studies have been carried out on its application to common Portland cement mortar. As such, the objective of this study is to evaluate the potential of sargassum ash as a mineral addition to partially replace fine aggregates in Portland cement mortar. Characterization of the raw materials was carried out through X-ray fluorescence spectroscopy, loss on ignition, particle size distribution, Brunauer–Emmett–Teller (BET) analysis, real density, X-ray diffraction, scanning electron microscopy, and dispersion spectroscopy of electrons. The mortars were prepared by partially replacing the fine aggregate (sand) with sargassum ash at 0%, 5%, 10%, and 20%. Mortar performance was evaluated through water absorption, apparent porosity, apparent specific mass, and compressive strength 7, 28, and 63 days after curing. Lastly, a life cycle assessment was conducted in accordance with ISO standards 14040:2006 and 14044:2006. The results showed that replacing sand with sargassum ash increases water absorption and apparent porosity, and decreases the apparent specific mass and compressive strength as replacement increases. Nevertheless, the compressive strength results after 63 days for 5 and 10% replacement did not differ statistically from reference values. The life cycle assessment indicated that mortars with partial replacement of sand by sargassum ash show positive environmental impacts when compared to reference values for most categories, regardless of the scenario analyzed, especially for mortar with 10% replacement. As such, the use of sargassum ash at 10% does not alter the mortar’s compressive strength values after 63 days, but does reduce its environmental impact. The application of this biomass in civil construction materials provides a destination for this algae, and that can be a solution to mitigate the social, environmental, and economic problems it has been causing.

## 1. Introduction

The Sargasso Sea occupies an area of approximately 4 million km^2^ in the northwestern region of the Atlantic Ocean and stands out for its great ecological relevance to the area’s ecosystem [1]. The pelagic sargassum biomass contributes to the biodiversity of the local ecosystem due to it providing shelter and food for different marine animals species, such as invertebrates, fish, turtles, and even birds. It also subsidizes nutrients that can serve as substrate for other species [1,2,3,4].

Nevertheless, there has been a proliferation of pelagic sargassum biomass in recent years, expanding its occurrence to new regions like West Africa, northern Brazil, the Caribbean, and the Gulf of Mexico [1,5,6,7,8]. This phenomenon has been observed since 2011 and has been met with several hypotheses, mainly the increase in ocean surface temperature and changes in sea currents [3,7,9,10,11,12,13,14,15].

This can lead to sargassum landfalls, which have a negative impact on several spheres such as economic activities related to tourism and fishing, the region’s ecosystem, and the health of the local population due to the release of toxic gases associated with its decomposition on beaches [13,16,17,18,19]

One way to mitigate the impacts caused by sargassum landfalls on the coast is to study alternatives for utilizing sargassum biomass [20]. Within this context, the review by Rossignolo et al., (2022) [7], identified potential applications involving the use of sargassum as a raw material in materials for civil construction, such as the use of ash in cementitious composites.

According to Amador-Castro et al., (2021) [20], the burning of this biomass for energy production presents an alternative destination for the material that runs aground on beaches. This process can generate a high ash content, which can reach up to approximately 50% of the dry *Sargassum natans* and *Sargassum fluitans* present in this pelagic sargassum biomass [21].

The ash from sargassum biomass is rich in mineral content, with a predominance of Ca, K, Na, S, Cl, and Mg [8,18]. The presence of these minerals may enable their application as filler in cementitious composites, which may contribute to matrix packaging and the nucleation effect [7,22,23].

The application of ashes from algae in cementitious materials has already been studied, such as in the research by Gupta et al., (2020) [24], where they evaluated the materials used in biofuel production. According to these authors, a 2% ash content in weight was incorporated into Portland cement, and the hydration, mechanical performance, and permeability of the cement mortars were evaluated. It is noteworthy that at the peak of hydration, mortar with ash presented superior results to the reference formulation. Additionally, the better packaging of the particles reduced the values of water absorption by capillarity.

Bilba et al., (2023) [3], presented a study seeking to evaluate the pozzolanic reactivity of sargassum ash in cementitious composites. Nevertheless, according to Chapelle, chemical composition and structural surface tests showed that sargassum ash does not qualify as a pozzolanic material. This encouraged the study to explore its use as a fine aggregate.

The Life Cycle Assessment (LCA) is a research tool aimed at evaluating alternatives for the use of sargassum biomass. It has tools that allow users to see an overview of the potential environmental impacts associated with the production systems of composite materials with the incorporation of algae. Bueno et al., (2023) [25], conducted an LCA study of different disposal scenarios for sargassum, including comparing different end-of-life alternatives and their respective potential impacts on landfilling; drying and processing fibers and/or particles for further use; and burning biomass for energy generation and ash production. For this research, an LCA evaluation and indicators of the ReCiPe 2016 methodology were used. The process of burning sargassum biomass presented a significant contribution from most of the indicators, mainly due to the considerable generation of particulates; however, the evaluation indicated a very significant reduction in the climate change emissions, from around 47% to less than 2%.

The aim of this study is to evaluate the potential of sargassum ash as a mineral addition to partially replace fine aggregate Portland cement mortars. Assessments on physical and mechanical properties were carried out, as well as an LCA study comparing the potential environmental impacts of mortar production systems with and without ash incorporation.

## 2. Materials and Methods

The experimental program involved physical, mechanical, and environmental tests in order to evaluate mortars with partial replacement of fine aggregates with sargassum ash.

**Figure 1:**
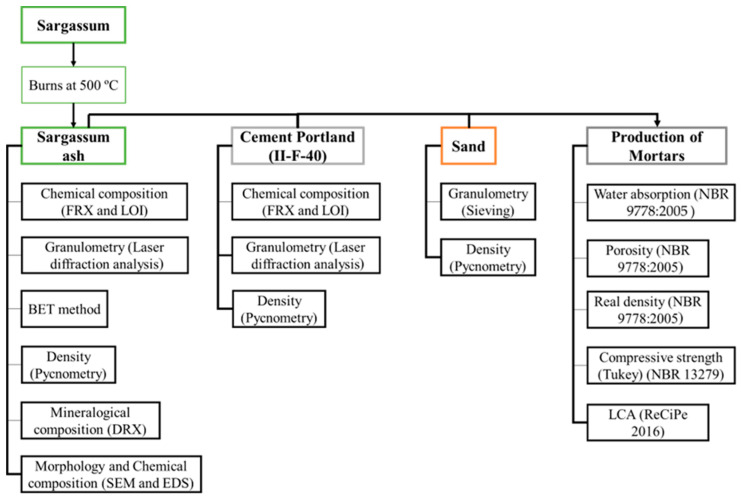
Diagram of the experimental stages in this research [26,27].

The raw materials were first characterized in order to understand their behavior when the mortars were produced. After production, the test samples were characterized, thus providing a view into the behavior of sargassum ash in cementitious composites. At the end, an LCA was carried out to verify the environmental viability of this new material. The experimental program was subdivided according to the diagram in Figure 1.

### 2.1. Experimental Procedure

#### 2.1.1. Characterization of Raw Materials

**Figure 2:**
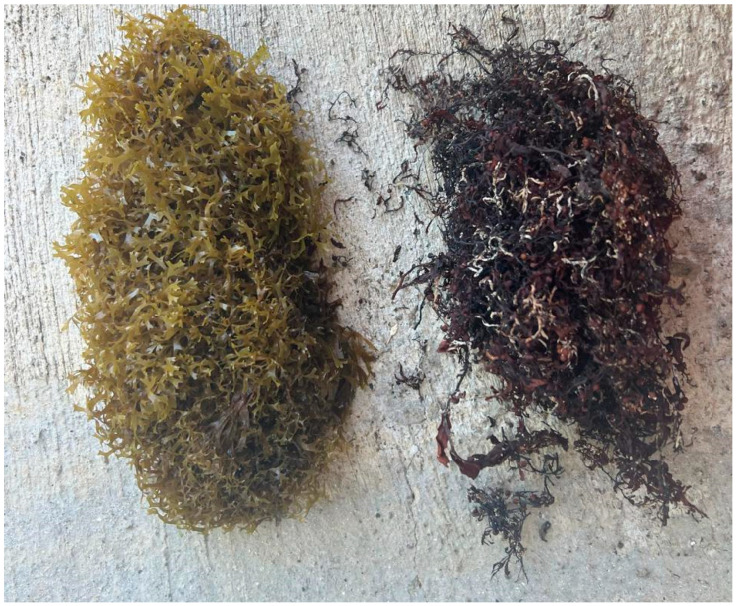
Sargassum used in the research. On the **left** we have fresh seaweed, and on the **right**, we have dry seaweed.

The sargassum biomass (Figure 2) used to produce ash was collected in the municipality of Carutapera-MA (Brazil), specifically in the region of São Pedro beach. According to Bueno et al., (2023) [25], the composition of the brown algae found in this region is as follows: *Sargassum fluitans* III (50%), *Sargassum natans* I (15%), and *Sargassum natans* VIII (35%).

The sargassum ash was obtained by means of a 10013 Jung resistive oven with 7000 W, a burning temperature of 500 °C for two hours, and a heating rate of 10 °C/min. The Portland cement (PC) was type II-F-40, corresponding to type II of the ASTM C150:2022 [28]. The natural fine aggregate used in this research came from the bed of the Mogi Guaçu River, located in the state of São Paulo, Brazil.

An X-ray fluorescence spectrometer (XRF) (PANalytical, Malvern, UK) and MiniPal4 model were used to determine the cement and sargassum ash chemical compositions through the determination of oxide values present in these materials (% by weight). Loss on ignition (LOI) was performed according to procedures prescribed in NBR NM 18: 2018 [29], to identify the amount of mass lost to a temperature of 1000 °C.

According to Chen et al., (2015) [30], the chemical composition of sargassum ash can vary according to the burning temperature, presenting refractory minerals at high temperatures, which can cause a change in the structure of the ash. Table 1 shows the chemical compositions of sargassum ash materials and CP II-F-40 cement, obtained from X-ray fluorescence.

The CaO, SO_3_, and Na_2_O oxide contents are the main constituents of sargassum ash, and their collective content is more than 58% of the total composition. The sargassum ash composition has values close to those of *Sargassum natans* ash, as shown by Chen et al., (2015) [30]. There are similar CaO values (28.11%), but lower SO_3_ values (4.07%), and higher Cl values (28.16%).

Each chemical element in Portland cement interferes with its composition in some way. According to Rojas and Cincotto, (2013) [31], the CaO levels (65.27%) are within the usual Portland cement composition limits (60–67%), while SiO_2_ content (14.26%) is below the usual (17–25%).

The granulometry of the raw materials, as well as that of the Portland cement and sargassum ash, was performed using a LA-950V2 Horiba laser particle size analyzer (HORIBA, Kyoto, Japan). The subsequent particle size distribution curves and the mean particle diameter values (Daverage, D10, D50, and D90) were analyzed. Table 2 shows the results of the sargassum ash particle sizes obtained by laser granulometry.

The diameter of 50% of the sargassum ash is 25.07 μm, while the cement had a diameter of 12.71 μm. The sand particle size distribution was carried out with sieves of different mesh sizes. Table 3 presents the results.

Due to the amount of material retained in each mesh, the sand used in this study was classified as fine in accordance with the NBR 7211:2022 standard [32]. Particle distribution influences paste and mortar properties in their fresh state, such as consistency and reactivity. In the hardened state, mechanical resistance and durability are assessed.

The surface area of the sargassum ash was determined though the BET method (Stephen Brunauer, Paul Hugh Emmett, and Edward Teller) using a Belsorp Max and MicrotracBEL gas adsorptometer (Microtrac, Osaka, Japan), with adsorption of N_2_ at 77K. The value was determined as 45.29 m^2^/g. The porosity in sargassum ash should increase the adsorption process, which is directly related to the surface area.

A quantity of the material (Portland cement, sargassum ash, and sand) was used for the density analysis. The results were obtained through a MVP-D160-E Quantachrome Multipycnometer (Quantachrome Instruments, Boynton Beach, FL, USA). This took the relationship between the mass of the solid and its actual volume into account (volume of the solid minus the volume of empty pores). The density of the CP II-F-40 cement was 3.05 g/cm^3^. The density found in the sargassum ash was 2.55 g/cm^3^, a result similar to that found by Bilba et al., (2023) [3]. Nevertheless, it is important to highlight that the actual density or specific mass may vary according to the sargassum collection site. The sand, a fine-grained aggregate, had a density value of 2.64 g/cm^3^. This is a density close to that of sargassum ash, thus allowing its replacement in mortars.

**Figure 3:**
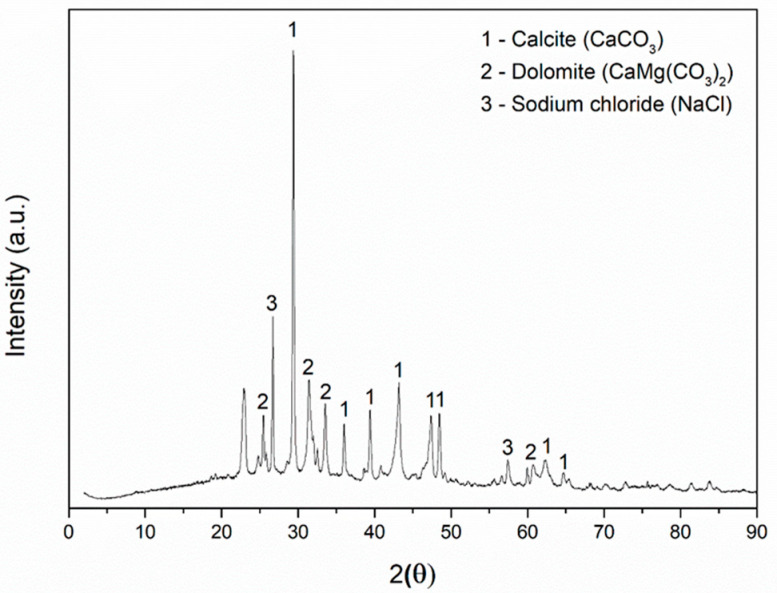
Diffractogram of the *Sargassum* spp. ash.

The mineralogical composition of the sargassum ash and cementitious pastes were determined by X-ray diffraction through a Rigaku Rotaflex Miniflex 600 (Rigaku, Tokyo, Japan) with CuKα radiation, a voltage of 40 kV, a 15 mA current, a scanning mode through steps of 0.02°, and an angle of 2θ traversed from 0° to 90°. Compounds already found in other studies from the literature—such as dolomite (CaMg(CO_3_)_2_) (JCPDS card 96-900-1298), calcite (CaCO_3_) (JCPDS card 00-005-0586), and halite (NaCl) (JCPDS card 00-005-0628) [33,34]—were identified in the sargassum ash X-ray diffraction analysis (Figure 3).

According to the analysis in Paraguay-Delgado et al., (2020) [33], calcite is present in all sargassum structures—both leaves and stems. Zhao et al., (2016) [35], observed that alkali metals in the form of phosphorus and sodium are organic at low temperatures. Nevertheless, they found that alkali metal chlorides were not eliminated, even at high combustion temperatures [35]. Halite (NaCl) and some unidentified impurities are observed. The crystalline halite phase is present in sargassum ash, as its intensity is reduced when the burning temperature increases [34].

The scanning electron micrograph (SEM) of the sargassum ash was obtained through a HITACHI TM 3000 scanning electron microscope (Hitachi High-Technologies Corporation, Tokyo, Japan). The sample was placed on a carbon ribbon and the photomicrograph was obtained by secondary electrons from the inner surface of the sargassum ash with an energy dispersive X-ray analyzer (EDS). As a result, the position of the peaks in the spectrum allow the identification of the elements present in the sargassum ash. The voltage for the EDS was 15 kV, and the counts were accumulated over 30 s.

**Figure 4:**
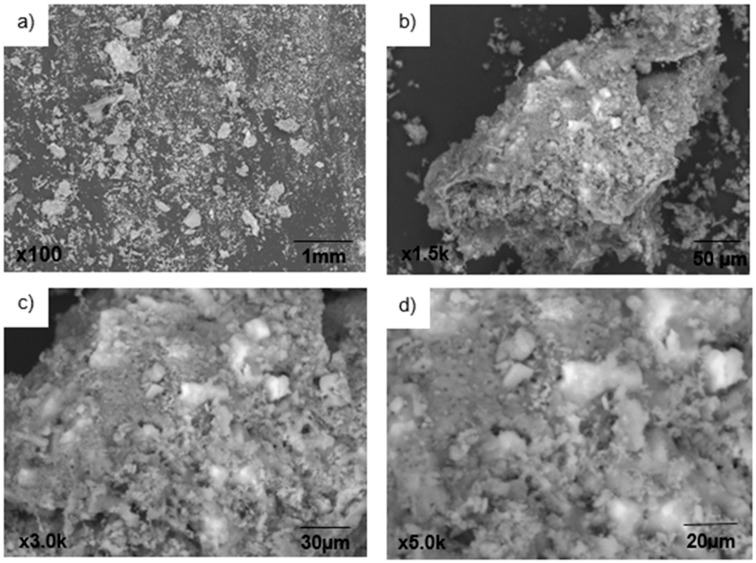
Micrographs of *Sargassum* spp. ash particles.

The presence of pores in the sargassum ash morphology was verified by SEM analyses. Microphotographs of the sargassum ash particles at different magnifications are shown in Figure 4.

The sargassum ash particle microstructures showed a variety in pore size, presence of roughness, and formation of structures, indicating heterogeneous morphologies. The higher the magnification (Figure 4a–d), the more the sharpness of the images is lost and shadows are formed due to the presence of very small clumps on their surface. According to Song et al., (2014) [34], the increase in roughness and the formation of a pore-like structure are attributed to the removal of inorganic impurities (mainly sodium, magnesium, and chloride). Nevertheless, in the research by Zhao et al., (2016) [35], there are images obtained by SEM for calcined sargassum ash samples at different temperatures (700 °C–900 °C), indicating a significant increase in porous structures as temperatures increase.

**Figure 5:**
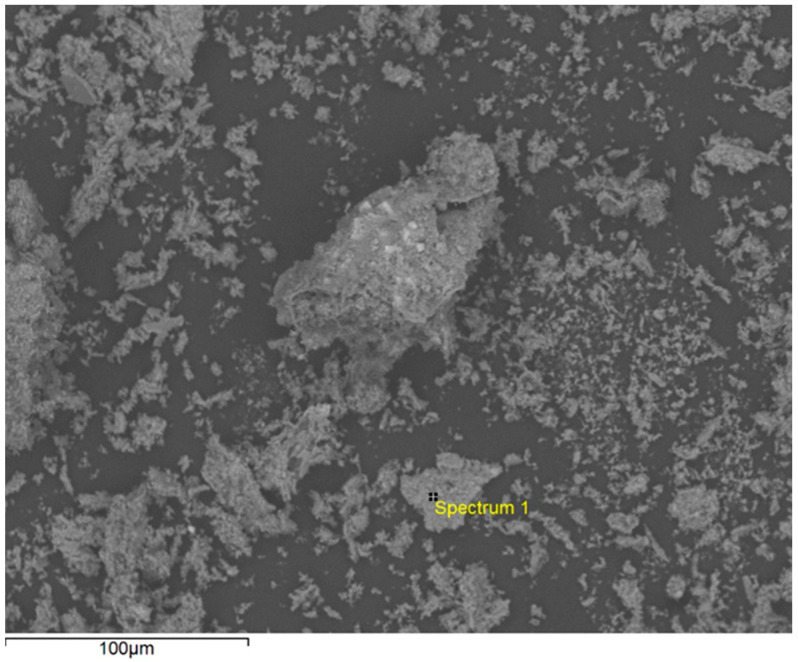
EDS analysis.

Microphotography was performed on a 322.2 μm particle of sargassum ash (Figure 5), but the percentage analysis of element concentrations was not performed.

**Figure 6:**
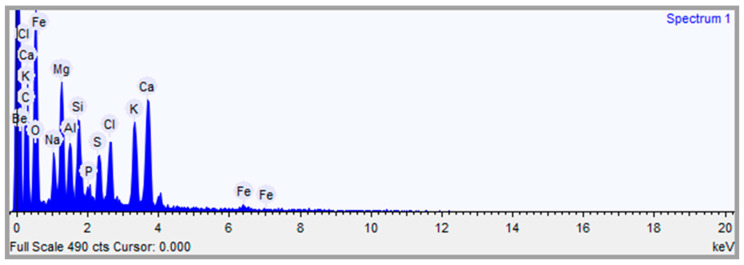
*Sargassum* spp. ash energy dispersive X-ray spectroscopy.

The sargassum ash EDS analysis confirmed the presence of the following elements: carbon (C), oxygen (O), sulfur (S), sodium (Na), magnesium (Mg), aluminum (Al), silicon (Si), chloride (Cl), potassium (K), calcium (Ca), beryllium (Be), and iron (Fe), as shown in Figure 6.

The chemical element composition of sargassum ash (Figure 6) resembles the phases of the compounds identified by X-ray diffraction and corresponds with the chemical elements present in the XRF results. The exception is the metallic component beryllium (Be), which may be related to metal adsorption by sargassum algae. Additionally, the EDS results are similar to those found in the research by López-Sosa et al., (2020) [36], due to the presence of the same chemical elements in sargassum stems and leaves from Mexico. The large amount of carbon, calcium, and oxygen elements may be related to the calcite phase [33].

#### 2.1.2. Production of the Mortars

In order to study Portland cement mortars, the formulation chosen as a reference is the same for conventional concrete (300 kg/m^3^) with a water/binder ratio of 0.5 (in mass) [37]. The binder/aggregate ratio was set at 1:2.5 in bulk.

Mortars were made with a 0% (M0SA), 5% (M5SA), 10% (M10SA), and 20% (M20SA) sargassum ash addition in relation to mass, as shown in Table 4.

The preparation of the mortars was done in an RV02E Eirich mixer. First, cement was added and mixed for 1 minute at slow speed; then sand, sargassum ash, and water were added for 2 minutes at slow speed, and subsequently mixed for 30 seconds. After that, the mixer was turned on at high speed for 2 minutes (Table 4).

Cylindrical samples 50 mm in diameter and 100 mm in height were molded for each mortar content series (0%, 5%, 10%, and 20%). The placement of the mortar mass in the cylindrical mold was carried out in two layers. The mold was filled and tapped on its surface in order to settle the mass. This mold was complemented with more mass until completely filled. The surface was then leveled with a trowel and taken to a vibrating table for 30 s in order to disperse air bubbles.

With the molds ready, these were wrapped in a plastic bag for 24 h for saturation. After this, they were removed from the cylindrical mold and placed in a container submerged in water for curing over 7, 28, and 63 days, respectively.

#### 2.1.3. Physical and Mechanical Characterization

The standard used for water absorption by total immersion was NBR 9778:2005 [26] in mortars with 28 days of curing. Three cylindrical samples were used in the water absorption process, where water is made to fill the permeable pores. The samples were kept in an oven at 105 °C for about 72 h, then had their mass recorded. These were subsequently immersed in water at room temperature—around 23 °C—and kept for 72 h. After this period, the saturated masses and submerged samples were determined with the aid of a hydrostatic scale. The water absorption and void index values of the mortars after 72 h were calculated along with the mass values obtained from the mortar samples, as well as the specific masses of the mortars in their different compositions (0%, 5%, 10%, and 20%).

**Figure 7:**
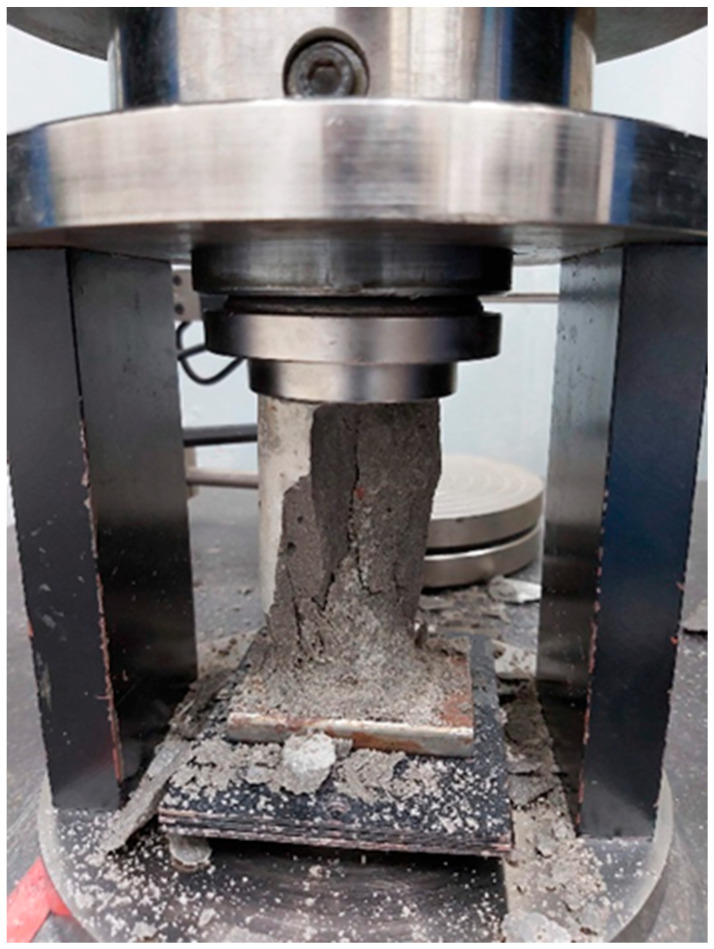
Cylindrical mortar samples after the compression test.

Fifteen cylindrical samples with a diameter of (50 ± 0.1) mm and a height of (100 ± 0.1) mm were used in order to measure the compressive strength of the mortars after 7, 28, and 63 days. The cylindrical samples were submerged in water before the compression test was performed. The mortars were subsequently compacted with an EMIC electromechanical press with a 100 kN load cell (Figure 7). This compression test followed the requirements of the NBR 13279 standard, applying a load (500 ± 50) N/s until the samples ruptured [27]. With the results of the compression tests, the final strength was obtained by arithmetic mean. The results were analyzed through a Tukey’s test with 5% significance.

### 2.2. Life Cycle Assessment (LCA)

#### 2.2.1. Goal and Scope

The objective of the LCA was to evaluate the environmental performance of mortars with partial replacement of fine aggregate sand with sargassum ash at different quantities.

Thus, LCA was applied through the following steps: definition of objective and scope; life cycle inventory analysis (LCI); life cycle impact assessment (LCIA); and interpretation of results. The methodology followed the ISO 14040 (2006) and ISO 14044 (2006) standards [38,39], thus identifying emissions from mortar production, their hot spots, and possible mitigations from the use of sargassum ash. Four different mortar compositions were evaluated according to Table 4.

Three different scenarios were evaluated and compared for the different mortar compositions.

1st scenario: all emissions from the mortar’s production process and its different compositions were considered, without taking into account any type of mitigation.

2nd scenario: the emissions from the process were considered, as well as the emissions avoided due to the use of sargassum ash, which prevented it from decomposing on the beach.

3rd scenario: emissions from the process were also considered, but in this case the avoided emissions due to the use of sargassum ash were taken into account, thus preventing it from decomposing in landfills.

**Figure 8:**
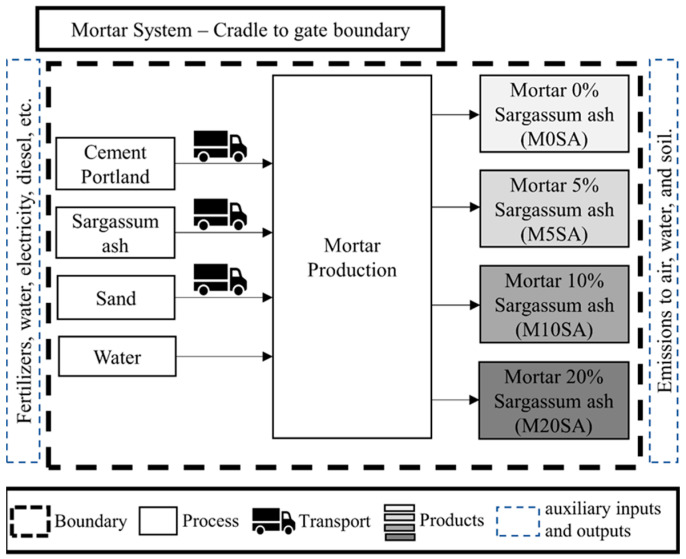
System boundaries of the different mortar compositions.

For mortars with sargassum ash, the system function is for application in projects with a minimum useful life of 50 years, according to NBR 15575-1 [40]. The functional unit was 1 m^3^ of mortar, which was used as a basis of comparison for this research. The cradle-to-gate system was adopted, which encompasses the acquisition of raw materials, the transport, and the production of mortars for use in civil construction (final product). The use and final disposal stages of the product were disregarded, as there are no LCA studies on mortars using sargassum ash. The limits of the product life cycle are illustrated in Figure 8.

**Figure 9:**
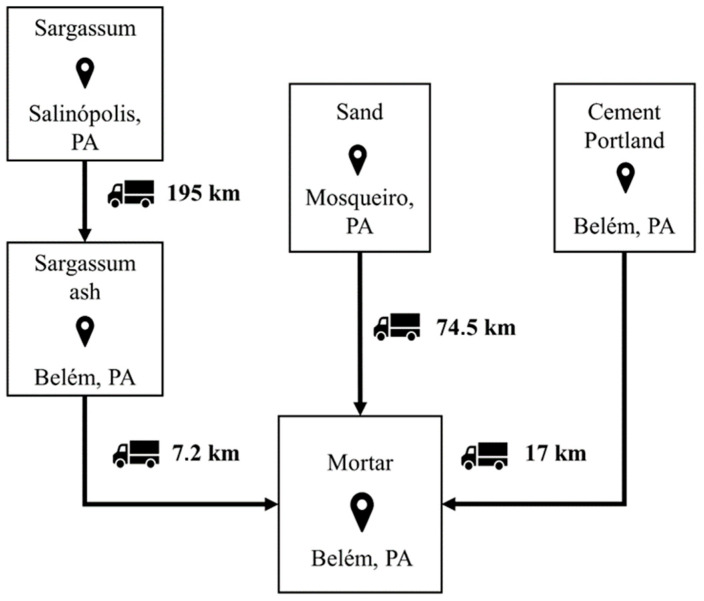
Geographic scope adopted in mortar production.

The production of mortar samples and physical–chemical tests were carried out in Pirassununga. Despite this, the geographic scope considered for the LCA was the city of Belém in the state of Pará, Brazil. The city of Belém was chosen because there are sargassum landfalls on the coast of Pará, facilitating the collection of this raw material due to shorter transport distances. Furthermore, the city of Belém has infrastructure that can absorb sargassum biomass for the generation of electrical energy, subsequently generating the ash for the production of mortars. This region also features cement and mortar-producing industries. There is also the city of Mosqueiro near the city of Belém, where sand deposits are located. If mortar production took place in regions far from the raw materials, the process would become unfeasible from an environmental and economic point of view. All process locations were based on actual facilities, and Figure 9 shows the geographic scope.

#### 2.2.2. Life Cycle Inventory (LCI)

Secondary data were used for the LCI. Information on sargassum ash, as well as its decomposition on beaches and landfills, was collected from the study by Bueno et al., (2023) [25]. Data relating to other processes were obtained from Ecoinvent 3.7.1. This database comprises average inventory data on materials and construction processes relevant in diverse regional contexts. Table 5 presents the secondary data used for mortar LCAs.

#### 2.2.3. Life Cycle Impact Assessment (LCIA)

Potential impact estimates were carried out with the LCIA methodology, specifically the ReCiPe 2016 method, through a hierarchical approach. The chosen method is one of the most recent, and despite being of European origin, it presents global data and was used as there is no Brazilian methodology. The inventory and calculations were carried out through Gabi 6.0 software. Nineteen different impact categories were evaluated in this study.

## 3. Results

### 3.1. Physical Properties

Table 6 shows the mortars’ mean water absorption values by immersion and the void index, according to the NBR 9778:2005 standard [26].

Mortar water absorption increases with the replacement of sargassum ash (0%, 5%, 10%, and 20%), and may be related to the increase in void rates. The higher the ash content (M20SA), the greater the porosity found in the mortars. Sargassum can generate capillary porosity and store water in these regions. The absorption of water by total immersion indicates the moment when mass becomes constant, no longer increasing in volume. This is correlated with the total porosity of the hardened mortar [42]. M20SA had an increase of 4.57% of the void index and absorbed 2.70% more water when compared to the reference mortar (M0SA). The void index is related to the microstructure and permeability of the samples, and the variation in water absorption values is directly linked to the void index.

The specific mass of each mortar decreased as the percentage of ash increased. This was due to the greater presence of porosity, which was observed in the void index values. Thus, M20SA showed higher water absorption and porosity, and lower specific mass values. We expected that the samples with ash would present lower specific masses, since sargassum ash has a lower real specific mass (2.55 g/cm^3^) than sand (2.64 g/cm^3^).

### 3.2. Mechanical Properties

**Figure 10:**
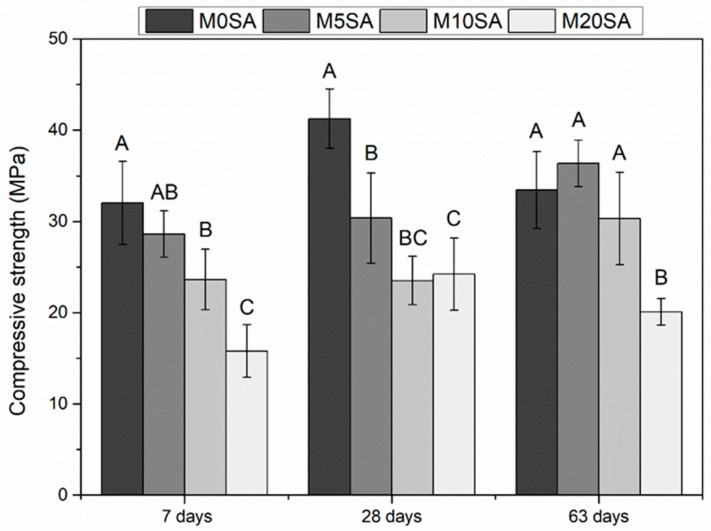
Compressive strength results of mortars after curing times of 7, 28, and 63 days. Averages followed by different letters in the columns are different according to the analysis of variance at a 5% significance level.

The effects of 0%, 5%, 10%, and 20% replacement of fine aggregate with curing times of 7, 28, and 63 days are shown in Figure 10. The lowest mortar strength value 7 days after curing was 20%, which explains why the increase in the proportion of sargassum ash reduces compressive strength. This result in M20SA correlates with its physical characteristics, data on water absorption by immersion, and porosity. Thus, the increase in pores can occasionally lead to greater fragility in the material, and consequently lead to a decrease in compression resistance. Furthermore, we must consider the possible interaction between the sargassum components and cement, since although sand is almost entirely composed of inert material, quartz and sargassum ash are not. This may influence the greater compression resistance presented by the M5SA and M10SA mortars at 63 days.

A Tukey’s test was performed to compare the average compressive strength values of the different treatments at a significance level of 5% for 7, 28, and 63 days of curing. We observed that all treatments showed differences between them for 7 and 28 days, with the reference mortar (M0SA) showing the best results (Figure 10). At 63 days, the M0SA, M5SA, and M10SA treatments did not present statistical differences. According to results found using the physical properties of M5SA and M10SA, 5% and 10% replacement are the ideal proportions (Table 6).

### 3.3. Life Cycle Assessment (LCA)

The LCIA methodology used in this ReCiPe 2016 study contains 19 impact categories. Table 7 presents the categories and their respective acronyms.

This item presents the comparative LCA of the four different mortars: M0SA, M5SA, M10SA, and M20SA.

Mitigations from the use of sargassum were not taken into account when analyzing the contribution of each process, except in the comparison stages of scenarios 2 and 3. This allows us to analyze which stage presents the most significant negative environmental impacts while suggesting solutions to mitigate them.

**Figure 11:**
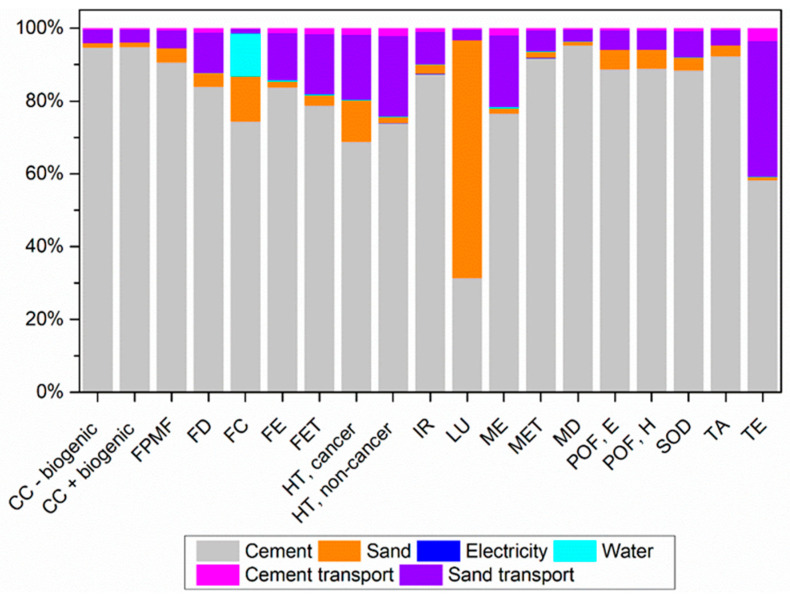
Percentage contributions of potential environmental impacts from mortar production with 0% replacement of sand with sargassum ash (M0SA), according to ReCiPe 2016.

Figure 11 depicts the environmental impact contributions from mortar production without the substitution of sand with sargassum ashes (M0SA).

Cement production is the stage with the greatest contribution to 18 impact categories among a total of 19. The majority of cement emissions occur due to the production of clinker, where raw materials are heated to approximately 1450 °C in rotary kilns using non-renewable fuels such as oil and coal. Additionally, there are considerable emissions from the transportation used throughout the production chain.

Climate change categories are among the most affected by cement production; it is estimated that for every 1 kg of cement, approximately 0.9 kg of CO_2_ is emitted due to the limestone calcination process.

For the Terrestrial Ecotoxicity category, the transport process brings a significant contribution, especially for sand, due to the use of fossil fuels such as diesel oil, and thus it ranks as the second-largest contribution.

In the Land Use category, sand extraction is the process that contributes the most due to the transformation, occupation, and clearing of the soil that occurs in extensive areas. Following that, cement production causes soil transformation due to the extraction of natural raw materials such as clay and lime.

**Figure 12:**
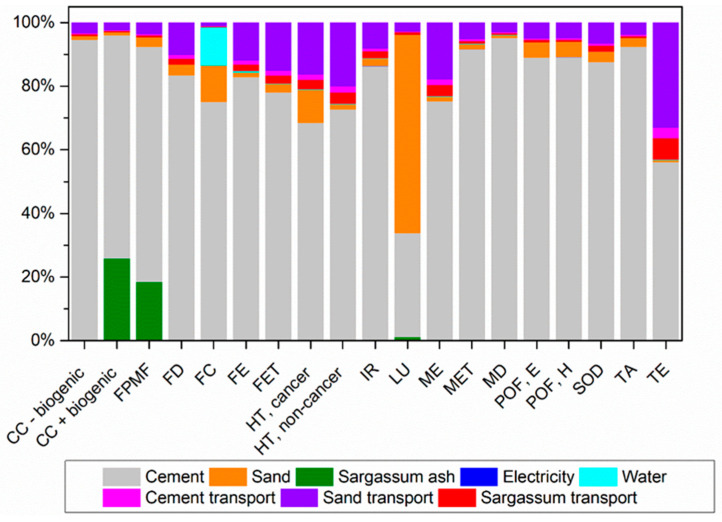
Percentage contributions of potential environmental impacts from the production of mortar with sand replaced with sargassum ash at 5% (M5SA), according to ReCiPe 2016.

**Figure 13:**
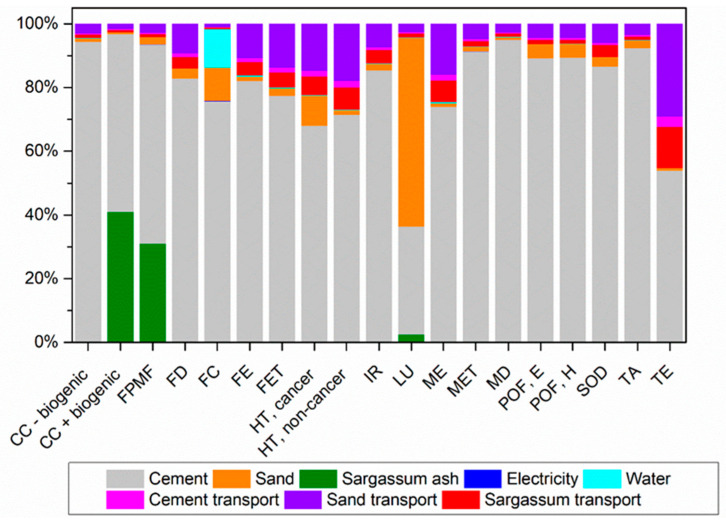
Percentage contributions of potential environmental impacts from the production of mortar with sand replaced with sargassum ash at 10% (M5SA), according to ReCiPe 2016.

**Figure 14:**
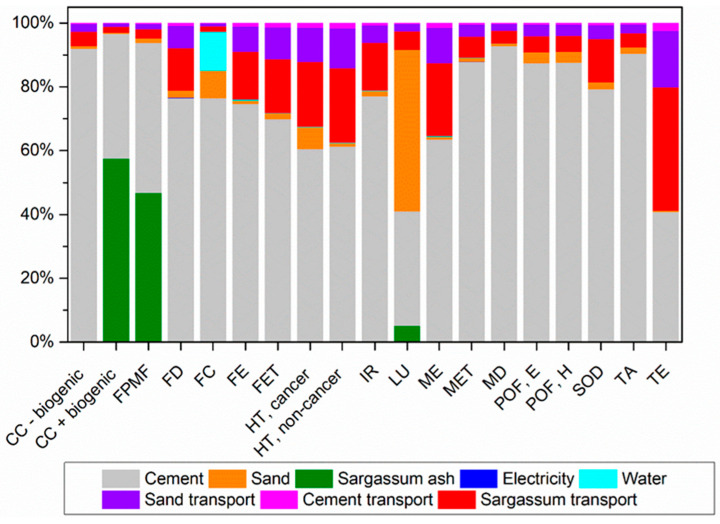
Percentage contributions of potential environmental impacts from the production of mortar with sand replaced with sargassum ash at 20% (M5SA), according to ReCiPe 2016.

Figure 12, Figure 13 and Figure 14 illustrate the contribution of each unit process to the potential environmental impacts of mortars with sand replaced with sargassum ash at 5%, 10%, and 20%, respectively.

There are no changes in the major processes contributing to the impact categories when partially replacing sand with sargassum ash at 5% and 10%. Nevertheless, there is a decrease in the contribution to some of these categories due to emissions from the transportation and burning of sargassum.

Three impact categories differ when comparing the former to the results for mortars with a 20% substitution. For the CC+ biogenic and FPMF categories, the production of sargassum ash is the process contributing the most due to the generation of gases and particle formation from burning.

For the Terrestrial Ecotoxicity category, transportation becomes the stage with the greatest contribution, surpassing cement. This shift is attributed to the greater contribution of sargassum transportation, which covers the longest distance when compared to other raw materials. This represents approximately 59% of emissions for this category, considering all transport.

**Figure 15:**
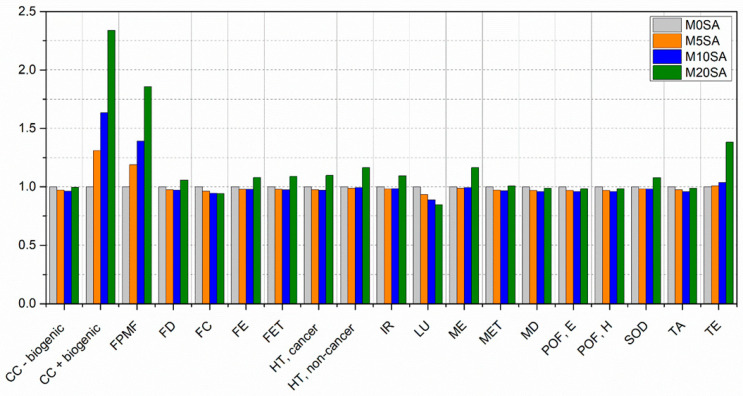
Comparison between the different scenarios of mortar with sand replaced by sargassum ash.

Figure 15 presents a comparison between the different mortar formulations with partial replacement of sand by sargassum ash, taking all process emissions without mitigation from the implementation of sargassum ash into account.

Mortar with 20% sargassum ash presented the worst performance for 12 of the 19 categories, while mortar without substitution performed the worst for 7 categories. The categories most affected by sargassum’s addition were CC + biogenic and FPMF, especially due to the processes of sargassum. The other categories in which sargassum presented the worst environmental performance were mainly affected by the transport distance.

The best environmental performance results were mostly attributed to the use of sargassum ash. Of the nineteen categories evaluated, replacing sand with sargassum showed the best performance for sixteen, including five categories at 5% substitution, nine categories at 10% substitution, and one at 20%. The mortar without sargassum ash showed the best results for three categories.

The application of 10% sargassum ash was excellent, with positive results for 9 categories and better results for 7 categories compared to the mortar without sargassum ash. The categories that showed the greatest discrepancy, with negative results for the application of 10% in relation to the reference, were CC + biogenic and FPMF. This is because the burning of sargassum has a considerable impact on the production of ash, in addition to the contribution due to the use of fuel to collect this material. These are points to be improved.

**Figure 16:**
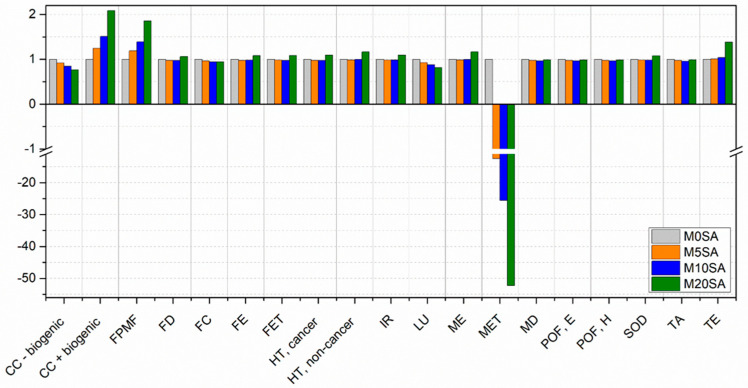
Comparison between the different scenarios of mortar with sand replaced by sargassum ash, considering the mitigation from sargassum decomposition on beaches.

**Figure 17:**
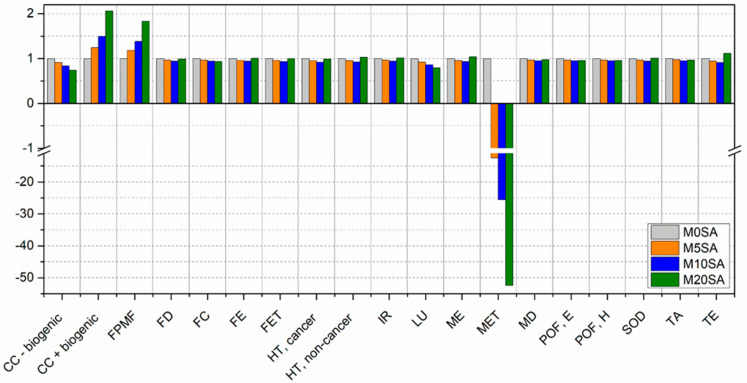
Comparison between the different scenarios of mortar with sand replaced by sargassum ash, considering the mitigation from sargassum decomposition in landfills.

Figure 16 and Figure 17 present the 2nd and 3rd scenarios presented in the methodology, where the mitigations for the non-decomposition of sargassum on the beach and in the landfill are considered, respectively.

Figure 16 presents a comparison between the different formulations with partial replacement of sand by sargassum ash while taking into account the mitigation of gases not emitted from decomposition on beaches.

The worst environmental performances are observed for the same formulations when mitigations are not considered, except for the Marine Eutrophication impact category. When taking the mitigation from avoiding its decomposition on beaches into account, mortar without sargassum ash presents the worst performance.

The best results undergo changes in three categories. Nevertheless, the use of sargassum ash still presents the best result for sixteen categories, including five categories for mortar with 5% replacement, seven categories for mortar with 10% replacement, and four categories for a 20% replacement.

The main change occurred in the Marine Eutrophication category and has a positive environmental impact when sargassum ash is used (as demonstrated in the graph in the negative results on the y-axis). The decomposition of sargassum on beaches increases nutrient levels in the sea, promoting its proliferation and affecting aquatic life. As such, highly positive impacts are observed for this category when this process is avoided.

Figure 17 presents a comparison between the different mortar formulations taking the mitigation of gases emitted from decomposition in landfills into account. This is a suitable assumption, as sargassum is typically removed from beaches and then taken to landfills in attempts to minimize its impact on tourism and maritime activities.

The decomposition of sargassum in landfills is the scenario that most closely resembles reality, as sargassum is currently removed from the coasts and sent to landfills due to its negative effects on local tourism.

When considering the mitigation of emissions from sargassum decomposition in landfills for the 19 categories, a 20% sargassum ash addition provided the worst environmental performance in 8 categories. For the other categories, mortars without sargassum ash presented the worst results.

The use of this ash in mortars showed a better environmental performance in 17 categories, including 13 for mortars with 10% replacement and 4 for a 20% replacement. Only CC + biogenic and FPMF presented the best results for mortar without the use of sargassum from the 19 categories; this is because the burning of sargassum generates gases and fine particles, which affect these 2 categories.

Among the categories, those that showed the most significant variations were Climate Change (including Biogenic Carbon), Fine Particulate Matter Formation, and Marine Eutrophication. Marine Eutrophication, as in the previous case, presents positive environmental impacts when sargassum ash is used.

Based on the results obtained for the different possibilities, it is possible to verify the importance of providing an adequate destination for sargassum, especially when taking mitigation from avoiding decomposition on beaches into account.

Therefore, the use of sargassum ash in mortars is a viable alternative, presenting better environmental performance results in addition to reducing the consumption of finite raw materials such as sand. As such, this destination for sargassum contributes to economic, social, and environmental aspects.

## 4. Conclusions

Several conclusions can be drawn from the experimental results obtained in this study, which included sargassum ash scanning electron microscopy and X-ray diffraction analyses, as well as mechanical and physical tests of the mortars.

Examination of sargassum ash SEM micrographs revealed a range of pore sizes, roughness, and varied structural formations, indicating a non-homogeneous morphology.

Replacing sand with sargassum ash led to increased water absorption and apparent porosity, along with a decrease in apparent specific mass values due to the capillary porosity and water retention properties of sargassum ash. Real specific mass values closely aligned with the density values of sargassum ash and sand determined by pycnometry tests.

The compressive strength of mortars decreased with higher levels of fine aggregate replaced by sargassum ash. Nevertheless, after 63 days, 5% and 10% additions showed statistically comparable results to the reference mortar. These percentages represent optimal replacements for non-structural applications like sealing blocks, plastering, and rendering, provided they meet regulatory standards for physical and mechanical properties.

Despite cement production being the primary contributor to the environmental impacts of all mortar compositions, the transportation of sargassum ash significantly contributed to various environmental impact categories. Hence, the use of sargassum ash in locations close to where it is sourced is recommended. Incorporating sargassum ash in mortars can mitigate environmental impacts, particularly when it prevents the decomposition of sargassum in landfills and reduces sand consumption.

The 10% replacement level exhibited compressive strength results statistically indistinguishable from the reference mortar and demonstrated superior environmental performance across different scenarios. Consequently, it can be concluded that the use of sargassum ash in mortars is feasible and aligns with the presented results. This approach aims to reduce raw material consumption through the application of sargassum algae byproducts, thereby fostering benefits within a circular economy framework.

This study underscores the potential of sargassum ash as a sustainable alternative in mortar production, offering technical viability as well as environmental benefits.

## Figures and Tables

**Table 1:** Chemical compositions of *Sargassum* spp. ash and Portland cement.

Oxides	*Sargassum* spp. Ash (%)	Portland Cement (%)
MgO	11.86	2.38
SiO_2_	7.13	14.26
P_2_O_5_	0.29	-
SO_3_	18.02	3.79
K_2_O	1.15	0.63
CaO	22.16	65.27
TiO_2_	0.43	030
Fe_2_O_3_	1.91	3.86
SrO	1.10	-
Cl	1.63	-
Br	0.52	-
Al_2_O_3_	2.58	2.28
In_2_O_3_	0.40	1.22
Na_2_O	17.72	-
LOI *	**13.00**	**6.00**

* LOI = loss on ignition.

**Table 2:** Particle size data of the *Sargassum* spp. ash and Portland cement.

Raw material	D_10_ (μm)	D_50_ (μm)	D_90_ (μm)
*Sargassum* spp. ash	9.59	25.07	66.78
Portland cement	3.94	12.71	24.15
D10—particle size below which 10% of the material is located; D50—particle size below which 50% of the material is located; D90—particle size below which 90% of the material is located.			

**Table 3:** Particle size data of the sand used in this experiment.

Sieve (mm)	% Average	% Accumulated
**9.50**	0	0
**6.30**	0.38	0.38
**4.00**	0.71	1.09
**1.00**	9.60	10.69
**0.50**	20.26	30.95
**0.10**	58.33	89.28
**Bottom**	10.72	100.00

**Table 4:** Mixing of mortars and their respective masses (%).

Mortar	Portland Cement	Sand	*Sargassum* spp. ash	Water
M0SA	25	62.5	-	12.5
M5SA	25	57.5	5	12.5
M10SA	25	52.5	10	12.5
M20SA	25	42.5	20	12.5

**Table 5:** Secondary data and respective sources used for mortar LCAs.

Process Inputs and Outputs	Data Source
***Input***	
**Materials**	Bueno et al., 2023 [25]
**Decomposition of sargassum on beaches**	
**Decomposition of sargassum in landfills**	
**Sargassum ash**	
**Portland Cement**, BR: Portland cement production	Ecoinvent 3.7.1
**Sand**, BR: sand quarry extraction operation	
**Water**, BR: tap water production, conventional treatment	
**Transport**	
**Transport**, RoW: transport, freight, lorry 16–32 metric ton, EURO 4 standard [41]	Ecoinvent 3.7.1
**Electricity consumption**	
**Electricity**, BR: electricity, high voltage, production mix	Ecoinvent 3.7.1
***Outputs***	
**Mortar**	-
**RoW—Rest of World; BR—Brazil**	

**Table 6:** Results by water absorption porosity and real density.

Mortar	Water Absorption (%)	Porosity (%)	Real Density (g/cm^3^)
**M0SA**	10.09	20.16	2.00
**S.D.**	0.29	0.58	0.00
**M5SA**	11.96	23.24	1.940
**S.D.**	0.09	0.20	0.01
**M10SA**	12.83	24.64	1.93
**S.D.**	0.04	0.06	0.01
**M20SA**	12.79	24.74	1.92
**S.D.**	0.07	0.31	0.01
**S.D.—Standard deviation**			

**Table 7:** ReCiPe 2016 impact categories and acronyms. Source: Bueno et al., 2023 [25].

Categories of Impact	Acronym
Climate Change, excl. Biogenic Carbon [kg CO_2_ eq.]	CC − biogenic
Climate Change, incl. Biogenic Carbon [kg CO_2_ eq.]	CC + biogenic
Fine Particulate Matter Formation [kg PM2.5 eq.]	FPMF
Fossil Depletion [kg oil eq.]	FD
Freshwater Consumption [m^3^]	FC
Freshwater Ecotoxicity [kg 1,4 DB eq.]	FE
Freshwater Eutrophication [kg P eq.]	FET
Human Toxicity, Cancer [kg 1,4-DB eq.]	HT, cancer
Human Toxicity, Non-Cancer [kg 1,4-DB eq.]	HT, non-cancer
Ionizing Radiation [kBq Co-60 eq. to air]	IR
Land Use [Annual crop eq.·y]	LU
Marine Ecotoxicity [kg 1,4-DB eq.]	ME
Marine Eutrophication [kg N eq.]	MET
Metal Depletion [kg Cu eq.]	MD
Photochemical Ozone Formation, Ecosystems [kg NO_x_ eq.]	POF, E
Photochemical Ozone Formation, Human Health [kg NO_x_ eq.]	PF, H
Stratospheric Ozone Depletion [kg CFC-11 eq.]	SOD
Terrestrial Acidification [kg SO_2_ eq.]	TA
Terrestrial Ecotoxicity [kg 1,4-DB eq.]	TE

## Data Availability

Data are contained within the article.
